# A Novel Intravascular Lithotripsy System in Severely Calcified Coronary Lesions: The Prospective COronary CAlcified Lesion Lithotripsy Procedure (COCALP) Study

**DOI:** 10.1002/mco2.70208

**Published:** 2025-05-20

**Authors:** Xin Deng, Yiqing Hu, Guosheng Fu, Genshan Ma, Xuebo Liu, Bei Shi, Jianfang Luo, Jingfeng Wang, Zhixiong Zhong, Hanbin Cui, Likun Ma, Juying Qian, Jian'an Wang, Hao Lu, Junbo Ge

**Affiliations:** ^1^ State Key Laboratory of Cardiovascular Diseases Department of Cardiology Zhongshan Hospital, Fudan University, Shanghai Institute of Cardiovascular Diseases; National Clinical Research Center for Interventional Medicine Shanghai China; ^2^ Department of Cardiology Key Laboratory of Cardiovascular Intervention and Regenerative Medicine of Zhejiang Province, Sir Run Run Shaw Hospital, Zhejiang University School of Medicine Hangzhou China; ^3^ Department of Cardiology Zhongda Hospital, Southeast University School of Medicine Nanjing China; ^4^ Department of Cardiology Shanghai Tongji Hospital, Tongji University School of Medicine Shanghai China; ^5^ Department of Cardiology Affiliated Hospital of Zunyi Medical University Zunyi China; ^6^ Department of Cardiology Guangdong Cardiovascular Institute, Guangdong Provincial People's Hospital, Guangdong Academy of Medical Sciences Guangzhou China; ^7^ Department of Cardiology Sun Yat‐sen Memorial Hospital, Sun Yat‐sen University, Guangzhou Key Laboratory of Molecular Mechanism and Translation in Major Cardiovascular Disease; Laboratory of Cardiac Electrophysiology and Arrhythmia in Guangdong Province Guangzhou China; ^8^ Center for Cardiovascular Diseases Meizhou People's Hospital (Huangtang Hospital), Meizhou Hospital Affiliated to Sun Yat‐sen University; Guangdong Provincial Engineering and Technology Research Center for Molecular Diagnostics of Cardiovascular Diseases; Guangdong Provincial Key Laboratory of Precision Medicine and Clinical Translational Research of Hakka Population Meizhou China; ^9^ Key Laboratory of Precision Medicine for Atherosclerotic Diseases of Zhejiang Province, The First Affiliated Hospital of Ningbo University; Cardiology Center, The First Affiliated Hospital of Ningbo University; Ningbo Clinical Research Center for Cardiovascular Disease Ningbo China; ^10^ Department of Cardiology The First Affiliated Hospital of USTC Division of Life Sciences and Medicine University of Science and Technology of China Hefei China; ^11^ Department of Cardiology The Second Affiliated Hospital of Zhejiang University School of Medicine, State Key Laboratory of Transvascular Implantation Devices, Heart Regeneration and Repair Key Laboratory of Zhejiang Province, Transvascular Implantation Devices Research Institute Hangzhou China

**Keywords:** effectiveness, intravascular lithotripsy, major adverse cardiovascular events, optical coherence tomography, safety, severe coronary calcification

## Abstract

Intravascular lithotripsy (IVL) is a promising therapy for calcified coronary lesions. This study evaluated the safety and effectiveness of a novel IVL system. The **CO**ronary **CA**lcified Lesion **L**ithotripsy **P**rocedure (COCALP) study (No. ChiCTR2300073280) was a prospective, multicenter, single‐arm trial involving 266 patients with severely calcified coronary lesions. The primary endpoint was procedural success, defined as successful stent implantation with ≤30% residual stenosis and no in‐hospital major adverse cardiovascular events (MACE). In a subgroup, calcium morphology was evaluated by optical coherence tomography (OCT) assessment. A total of 266 patients were included. The procedural success rate was 97.4% (95% confidence interval [CI]: 0.947–0.989), with the lower limit of the CI exceeding the prespecified performance goal (*p* < 0.001). No MACE occurred intraoperatively. During hospitalization, MACE occurred in five patients (1.9%), all of which were myocardial infarctions. MACE rates at 1 and 6 months were 2.3 and 3.4%, respectively. In the OCT subgroup (*n* = 76), IVL induced a 76.8% rate of calcification fracture. The minimal lumen area increased from 1.77 ± 0.72 to 2.59 ± 1.11 mm^2^ following IVL (*p *< 0.001), and further expanded to 5.22 ± 1.69 mm^2^ poststenting (*p *< 0.001). The novel IVL system demonstrated high effectiveness and safety, supporting its use for treating severely calcified coronary lesions and enhancing stent implantation success.

## Graphical abstract

***Safety and effectiveness of the novel intravascular lithotripsy (IVL) device***: The overall success rate of the novel intravascular lithotripsy (IVL) device (A) is 97.4%, with no significant differences observed across subgroups (age, gender, hypertensive, bifurcation, lesion length and lumen narrowing) (B). Additionally, optical coherence tomography (OCT) shows that stent implantation following IVL can achieve significant lumen gain (C).

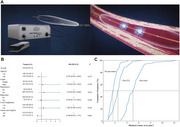

## 1 Introduction

Coronary artery disease (CAD) is the most prevalent form of panvascular disorders [1, 2, 3, 4, 5], and percutaneous coronary intervention (PCI) has witnessed remarkable progress over the years. Nevertheless, PCI continues to grapple with three major challenges: complex calcified lesions, often described as a tightening curse due to their persistent difficulty [6, 7]; chronic total occlusions (CTOs), widely regarded as the final frontier of PCI owing to their technical complexity [8, 9]; and in‐stent restenosis, considered the Achilles’ heel of PCI for its enduring impact on outcomes [10, 11]. According to the *Report on Cardiovascular Health and Diseases in China 2023*, the current prevalence of CAD in China is 11.39 million and 1.421 million patients underwent PCI in 2022 [12, 13]. Calcified coronary lesions present significant challenges in the cardiac catheterization laboratory, recognized in approximately 33% of patients undergoing PCI [14]. A patient‐level pooled analysis of seven contemporary stent trials reveals that approximately 20% of patients have severe calcification [15]. Meanwhile, among patients with acute coronary syndrome, around 27–38% exhibit one or more moderate to severely calcified target lesions [14].

Coronary calcification is primarily caused by the accumulation of calcified plaques within the arterial walls, leading to luminal narrowing. These calcified plaques are encapsulated by endothelial cells and the internal elastic membrane. The calcium content within the plaques hardens the stenotic vessels, impeding effective vascular dilation therapies. Calcified plaques impede the delivery of balloons and stents and increase the risk of inadequate stent expansion and malapposition [16, 17]. Additionally, calcified plaques act as a barrier, hindering the delivery of drugs from balloons or stent coatings to the target cells within the vascular media [18]. Moreover, aggressively advancing drug‐eluting stents through severely calcified lesions carries the risk of damaging the drug coating. Therefore, these lesions are notably associated with a higher incidence of stent failure, vascular complications—including dissection, perforation, and myocardial infarction (MI)—and major adverse cardiovascular events (MACE), which encompass death, MI, and target vessel revascularization [19, 20, 21].

Treatment strategies for calcified coronary lesions include the use of compliant, noncompliant, and cutting balloons [14]. Noncompliant balloons are effective in calcified lesions, especially at ostial locations, and they mitigate the risks of barotrauma, dissection, and perforation [22]. However, these devices remain associated with procedural failures. Severely calcified lesions are often difficult to fully address with balloon angioplasty, even when using noncompliant (high‐pressure) balloons [23]. Balloons tend to undergo asymmetric expansion and the “dog‐boning” effect at heavily calcified sites. This tendency to expand more in noncalcified areas increases the risk of dissections and perforations at the interface between calcified and healthy tissue [24, 25]. Semi‐compliant balloons, though often employed, carry similar risks. Consequently, semi‐ and noncompliant balloons are generally reserved for less severely calcified lesions. Cutting balloons, which are a type of noncompliant balloon equipped with surgical blades, facilitate the creation of small fractures in the calcium deposits, thereby reducing elastic recoil after balloon deflation. However, they have not demonstrated a significant reduction in restenosis and MACE rates compared with conventional noncompliant balloon angioplasty [26, 27]. Ablative techniques such as rotational, excimer laser, and orbital atherectomy, which pulverize calcium while minimizing interaction with elastic tissues, offer another treatment modality. Nevertheless, these techniques are not universally available and require a steep learning curve, and induce risks such as microcirculatory obstruction from calcium debris [28, 29]. Rotational atherectomy is currently the preferred treatment for severe calcification. It works by reducing plaques and calcified lesions into tiny particles (98% of which are smaller than 10 µm, with an average diameter of 5 µm—smaller than a mature red blood cell). These particles enter the capillary circulation, where they are believed to be cleared by the reticuloendothelial system [30, 31, 32]. However, if the particles generated by rotational atherectomy are larger (e.g., due to excessive deceleration), they can cause microvascular obstruction, leading to reduced contractility of the supported myocardium, slow flow/no‐reflow, and MI [33]. Additionally, the high‐speed rotation of the burr can cause thermal injury and activate platelets, thereby increasing the risk of thrombus formation [33]. Complications of rotational atherectomy include slow flow (2.5%), dissections (1.7–5.9%), and perforations (2%) [34].

Intravascular lithotripsy (IVL) has emerged as a novel and promising modality for the treatment of calcified coronary lesions, demonstrating both effectiveness and safety [35, 36, 37]. This technique utilizes electrohydraulic‐generated sonic pressure waves to disrupt subendothelial calcification and calcified nodules, thereby facilitating significant lumen area gain and optimal stent expansion [35]. Studies have highlighted that the modification of calcified plaques through fracture mechanisms is a key contributor to these favorable outcomes. For instance, the DISRUPT CAD trial series (I–IV) has consistently validated the safety of IVL, particularly in terms of 30‐day MACE, while also provided its effectiveness in aiding stent implantation [36, 37, 38, 39]. Beyond its safety profile, IVL has the unique ability to restore vascular elasticity, remodel blood flow in diseased vessels, and preserve endothelial integrity, offering advantages over other conventional therapies [40]. The SHOCKWAVE C2 IVL system has gained approval in many countries, underscoring its growing acceptance in the treatment of calcified coronary lesions. Notably, the recent CALCI‐CRACK trial conducted in China provided further evidence of IVL's effectiveness and safety in managing complex calcified coronary lesions, including true bifurcation lesions, severely tortuous vessels, and CTOs [41]. Despite these advancements, the clinical application and accessibility of IVL remain limited, particularly among Chinese patients. There is a pressing need for the continued development of new IVL systems to expand its availability and optimize outcomes in this patient population.

**FIGURE 1:**
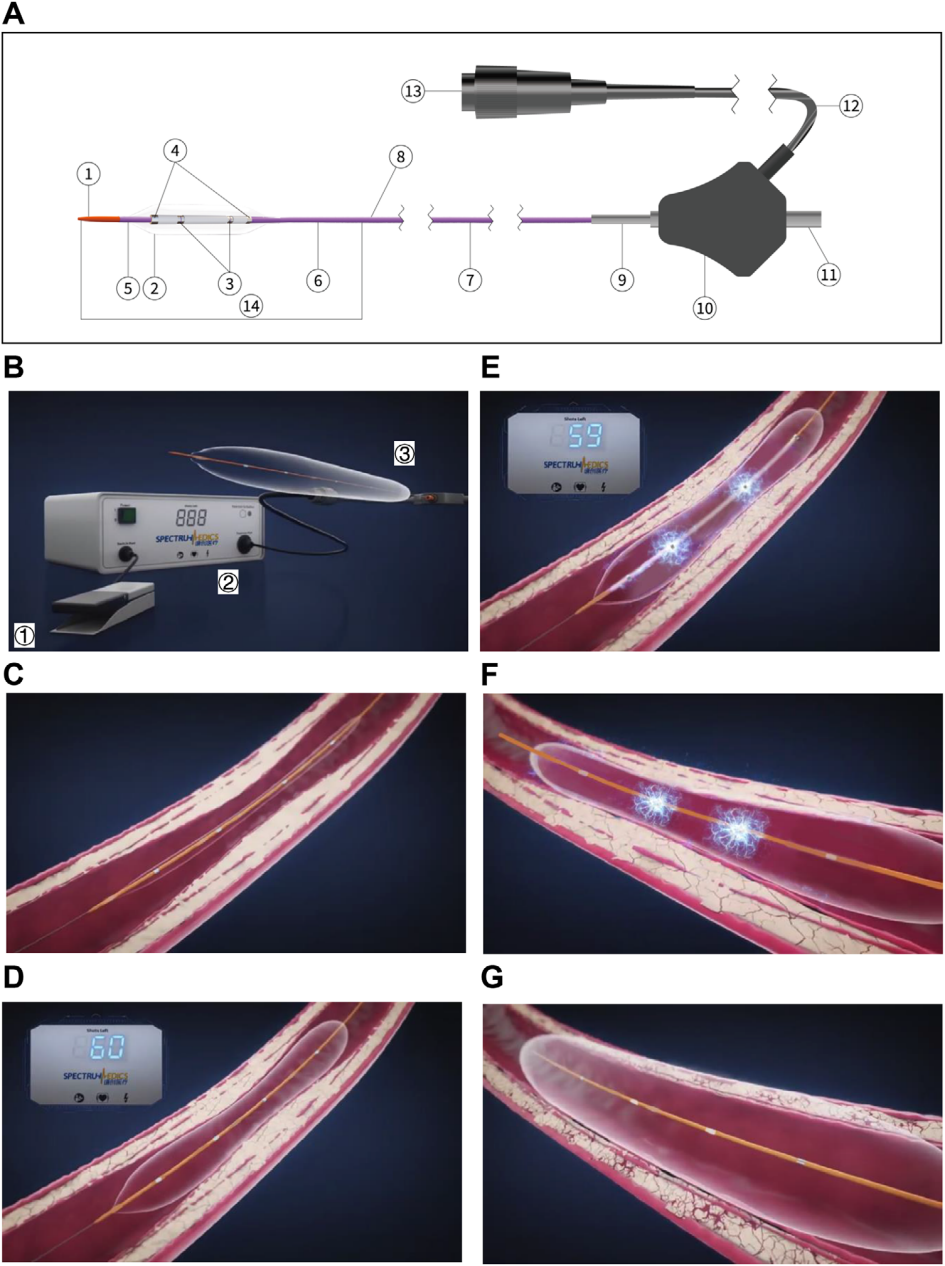
Structure and working principle of the Sonico‐CX coronary intravascular lithotripsy (IVL) balloon catheter. (A) Structure of the IVL balloon catheter: ① Distal tip, ② balloon, ③ emitter, ④ radiopaque marker, ⑤ inner tube, ⑥ distal outer tube, ⑦ proximal outer tube, ⑧ guidewire port, ⑨ stress diffusion tube, ⑩ housing assembly, ⑪ balloon inflation port, ⑫ device connection cable, ⑬ device connection interface, ⑭ hydrophilic coating. The IVL balloon catheter adopts a rapid exchange (Rx) design, allowing the inner tube to accommodate a 0.014‐in. guidewire. Radiopaque metal markers are located at both ends of the balloon's effective working length for fluoroscopic positioning. The lithotripsy emitters are positioned between the two markers. (B) Structure of the IVL system: ① Footswitch: frees the operator's hands. ② Intravascular lithotripsy generator: Automatically identifies the catheter model. The shockwave energy and pulse count are automatically adjusted by the generator's software, requiring no manual settings for ease of use. ③ IVL balloon catheter: Features a standard Rx balloon catheter design with two pairs of miniature lithotripsy emitters; delivers 360° circumferential shockwaves to uniformly fracture calcified rings. (C–G) Working principle of the IVL balloon: The shockwave catheter is advanced over the guidewire through the calcified lesion (C). The balloon is inflated to 4 atm to appose the vessel wall (D), and shockwave therapy is initiated (E). An electrical signal is transmitted through the conductive wire to the miniature electrodes within the balloon, generating a spark discharge in the electrolyte solution (a 1:1 mixture of 0.9% sodium chloride and contrast medium). This instantaneously vaporizes the solution, creating plasma. The plasma generates rapidly expanding and collapsing bubbles on the electrode surface, producing mechanical shockwaves (nonfocused) within the balloon. These shockwaves can directly penetrate the low‐density balloon and vascular soft tissue. However, when encountering high‐density, high‐acoustic impedance materials such as calcified plaques, the shockwave energy fractures them, restoring vessel compliance while leaving the calcified plaque in place (F). The balloon is then inflated at low pressure (8 atm) to further expand the lesion and increase the lumen (G), enabling subsequent stent implantation. IVL, intravascular lithotripsy.

A novel IVL system has recently been developed by Spectrumedics Medical Technology (Shanghai) Co., Ltd. (Shanghai, China) [42]. This advanced system includes the Sonico‐CX disposable intravascular hydroelectric shock wave balloon catheter, paired with a dedicated hydroelectric shock wave lithotripsy generator. The Sonico‐CX catheter features a reduced folded outer diameter (0.042∖0.044∖0.046 in.) and is available in seven sizes (2.5∖2.75∖3∖3.25∖3.5∖3.75∖4 mm), enabling precise adaptation to diverse coronary artery dimensions and optimal balloon apposition against the vessel wall. Equipped with two pairs of emitters, the catheter delivers consistent shockwave energy with 360° circumferential coverage, supporting up to 120 pulses per catheter (Figure 1)—significantly higher than the 80‐pulse limit of the SHOCKWAVE C2 IVL system (Shockwave Medical, Inc., Santa Clara, CA, USA) (Table S1). This innovation may enhance calcified lesion fragmentation and improve procedural outcomes in patients with severe coronary calcification.

Our previous study compared IVL and plain old balloon angioplasty in healthy porcine coronary arteries, finding no significant difference in intimal hyperplasia or endothelial damage [7]. This study was designed with robust statistical power to rigorously assess the effectiveness and safety of this novel IVL system in facilitating stent deployment in patients with severely calcified coronary lesions.

## 2 Results

### 2.1 Participants Characteristics

A total of 266 participants were enrolled, and included in both full analysis set (FAS) and safety set (SS). The per protocol set (PPS) included 249 participants. The baseline characteristics of the 266 participants, with a mean age of 69.2 ± 7.8 years and 62% being male, are detailed in Table 1. Nine discontinued the trial due to adverse events (AEs) (*n* = 6), loss to follow‐up (*n* = 2), or other reasons (*n* = 1), with 257 participants completing the study. The majority of participants (90.9%) presented with stable or unstable angina, which was the most common indication for PCI. Hypertension was prevalent, affecting 79.0% of the cohort.

**TABLE 1:** Characteristics of the participants (FAS).

Characteristics	Value (*n* = 266)
Age, years	69.2 ± 7.8
Male, *n* (%)	165 (62.0)
Hypertension, *n* (%)	210 (79.0)
Diabetes, *n (%)*	96 (36.1)
Atrial fibrillation, *n (%)*	9 (3.38)
Prior myocardial infarction within 30 days, *n* (%)	1 (0.4)
Prior transient ischemia attack or stroke within 3 months, *n* (%)	1 (0.4)
BMI (kg/m^2^) (*n* = 257)	23.7 ± 3.3
Serum creatinine (µmol/L) (*n* = 265)	81.3 ± 34.5
Low‐density lipoprotein‐cholesterol	1.99 ± 0.90
NYHA class	
I, *n* (%)	119 (44.7)
II, *n* (%)	144 (54.1)
III, *n* (%)	3 (1.1)
IV, *n* (%)	0
Left ventricular ejection fraction, % (*n* = 229)	63.4 ± 8.0
Angina assessment (*n* = 265)	
None, *n* (%)	24 (9.1)
Stable angina, *n* (%)	97 (36.6)
Unstable angina, *n* (%)	144 (54.3)

The number of patients in each statistical analysis was 266 if not indicated specifically.

The numbers following the symbol “±” indicate SD (standard deviation).

*Abbreviation*: FAS, full analysis set.

### 2.2 Angiographic Characteristics

As shown in Table S2, the left anterior descending artery (LAD) was the most frequently targeted vessel for IVL, accounting for 73.1% of cases. The minimum vessel diameter was 0.89 ± 0.36 mm, with a reference vessel diameter of 2.82 ± 0.38 mm. Stenosis averaged 68.7 ± 11.5%. The lesions averaged 35.0 ± 15.6 mm in length. Notably, 95.8% of lesions were eccentric, 10.2% exhibited tortuosity, and 27.3% involved bifurcations.

### 2.3 Procedural Characteristics

As detailed in Table S3, radial access was utilized in 94.7% of cases, and 6F guiding catheters were employed in 91.4% of procedures. The lithotripsy balloon had a mean diameter of 2.81 ± 0.33 mm, and the mean number of IVL pulses administered was 39.9 ± 23.3. Optical coherence tomography (OCT) data were available in 76 patients (28.6%) to assess coronary calcification. The stents implanted had a mean length of 40.6 ± 15.6 mm and a mean diameter of 2.91 ± 0.32 mm.

### 2.4 Effectiveness of IVL

A total of 302 Sonico‐CX catheters were used among the 266 participants. Four catheters failed to cross the calcification lesion, with a crossing rate of 98.68%. In FAS, two patients did not have data available for the primary effectiveness endpoint; one did not receive a stent, and the other withdrew from the clinical trial. For these patients, the primary endpoint was classified as a failure. As shown in Table 2, in‐stent residual stenosis ≤30% was achieved in all the other 264 patients with evaluable data (100%). The incidence of in‐hospital MACE was 1.9%, encompassing five MIs (all Type 4a). Consequently, procedural success was achieved in 259 out of 266 patients, reflecting a success rate of 97.4% (95% confidence interval [CI]: 0.947–0.989), with the lower bound of the 95% CI exceeding the prespecified goal (*p* < 0.001). In PPS, the procedural success rate was 98.0% (95% CI: 0.954–0.993), with the lower bound also significantly higher than performance goal (PG) (*p *< 0.001).

**TABLE 2:** Primary and secondary effectiveness endpoints.

Endpoints	*n* (%)
Procedural success	259 (97.4)
In‐stent residual stenosis ≤30%	264 (100)^a^
In‐hospital MACE	5 (1.9)
Angiographic success	261 (98.9)
In‐stent residual stenosis ≤30%	264 (100)^a^
Severe angiographic complications	3 (1.1)
Device success rate^b^	294 (97.4)

*Abbreviation*: MACE, major adverse cardiovascular events.

^a^ Two patients did not have data available for the primary effectiveness endpoint (one withdrew intraoperatively, and the other did not receive stent implantation), were not included in this analysis.

^b^ Count and percentage were calculated with number of balloon catheter used (*n* = 302).

**FIGURE 2:**
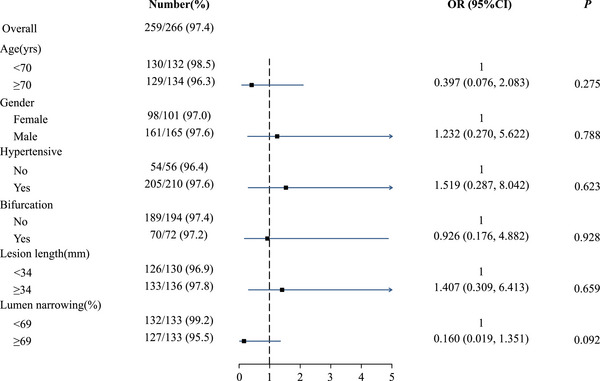
Subgroup analyses of procedural success (FAS). *p* Values were obtained via logistic regression to indicated the subgroups that were more likely to achieve procedural success. CI, confidence interval; FAS, full analysis set; OR, odds ratio.

Subgroup analyses, illustrated in Figure 2, revealed a consistently high procedural success rate across various subgroups, all exceeding 95%. The highest success rate, 99.2%, was observed in patients with lumen narrowing of less than 69%.

In FAS, three patients (1.1%) developed serious angiographic complications immediately following IVL, including one patient with acute occlusion, type F dissection and no‐reflow, one with slow‐flow, and another case of type F dissection. Thus, the angiographic success rate was 98.9%. The device success rate was 97.4%. In PPS, the rates of angiographic success and the device success were 98.8 and 98.6%, respectively.

### 2.5 Safety

As shown in Table 3, no MACE occurred intraoperatively. The incidence of in‐hospital MACE was 1.9% (five out of 266), with five MIs (all Type 4a). An additional MACE event was reported within the 30 days post IVL, with total number of MACE cases of six (2.3%). Within 6 months following the procedure, a cumulative total of nine patients experienced MACE, corresponding to a 3.4% incidence rate. All MACE were considered unrelated with investigational device.

**TABLE 3:** Safety endpoints.

Endpoints	*n* (%) (*n* = 266)
Intraoperative MACE	0
In‐hospital MACE	5 (1.9)
Cardiac death	0
Target vessel revascularization	0
Myocardial infarction	5 (1.9)
30‐day MACE	6 (2.3)
Cardiac death	1 (0.8)
Target vessel revascularization	0
Myocardial infarction	5 (1.9)
6‐month MACE	9 (3.4)
Cardiac death	4 (1.5)
Target vessel revascularization	0
Myocardial infarction	5 (1.9)
Any AE	157 (59.0)
Related to the IVL device	15 (5.6)
AEs leading to withdraw	6 (2.3)
Related to the IVL device	0
Serious AE	30 (11.3)
Related to the IVL device	0

*Abbreviations*: AE: adverse event; IVL: intravascular lithotripsy; MACE: major adverse cardiovascular events.

At 6‐month follow‐up, four cardiac deaths (1.5%) occurred (Table 3), with heterogeneous etiologies. One patient died suddenly 3 days after PCI (after discharge), presenting with syncope and cardiac arrest; premature discontinuation of dual antiplatelet therapy raised suspicion of stent thrombosis, though angiographic confirmation was unavailable. Another death resulted from intracranial hemorrhage 4 months post‐PCI, deemed unrelated to the procedure and attributed to underlying cerebrovascular disease. The remaining two deaths were linked to progressive heart failure—one occurring 2 months post‐PCI following an acute decompensation episode in a patient with multivessel disease and renal insufficiency, and the other 6 weeks after intervention in an elderly patient with advanced ischemic cardiomyopathy. Only the first case suggested possible stent thrombosis (Type 4b MI per Universal Definition), while others reflected either comorbid conditions or procedural complications unrelated to the device. Cause‐of‐death adjudication was limited by incomplete postmortem data in the suspected thrombotic case.

Serious angiographic complications occurred in three patients (0.7%), as described in the effectiveness endpoint of angiographic success. There was a case with acute occlusion, type F dissection and no‐reflow, which were resolved with administration of verapamil, nitroglycerin, stent implantation and balloon dilatation. Another case of type F dissection was also successfully managed with appropriate balloon dilatation and stent implantation. All serious angiographic complications were resolved following stent implantation. Additionally, one patient developed a poststent vessel complication, specifically a perforation (Ellis II) at the middle segment of the right coronary artery. The pericardial effusion due to perforation was managed through pericardiocentesis and drainage. The perforation was controlled by balloon occlusion and stent implantation, and subsequent angiography showed no evidence of contrast extravasation. Follow‐up echocardiography revealed no pericardial effusion, and the pericardial drain showed no further hemorrhagic fluid. This case of perforation was considered attributed to guidewire mismanagement in the middle segment of the right coronary artery and was not related to IVL performed on the target vessel.

A total of 157 (59.0%) patients had AEs, which were considered related with the IVL device in 15 cases (5.6%). The incidence of serious AE (SAE) was 11.3% (30 out of 266), none of which was related with the investigational device.

### 2.6 OCT Subgroup Analysis

**FIGURE 3:**
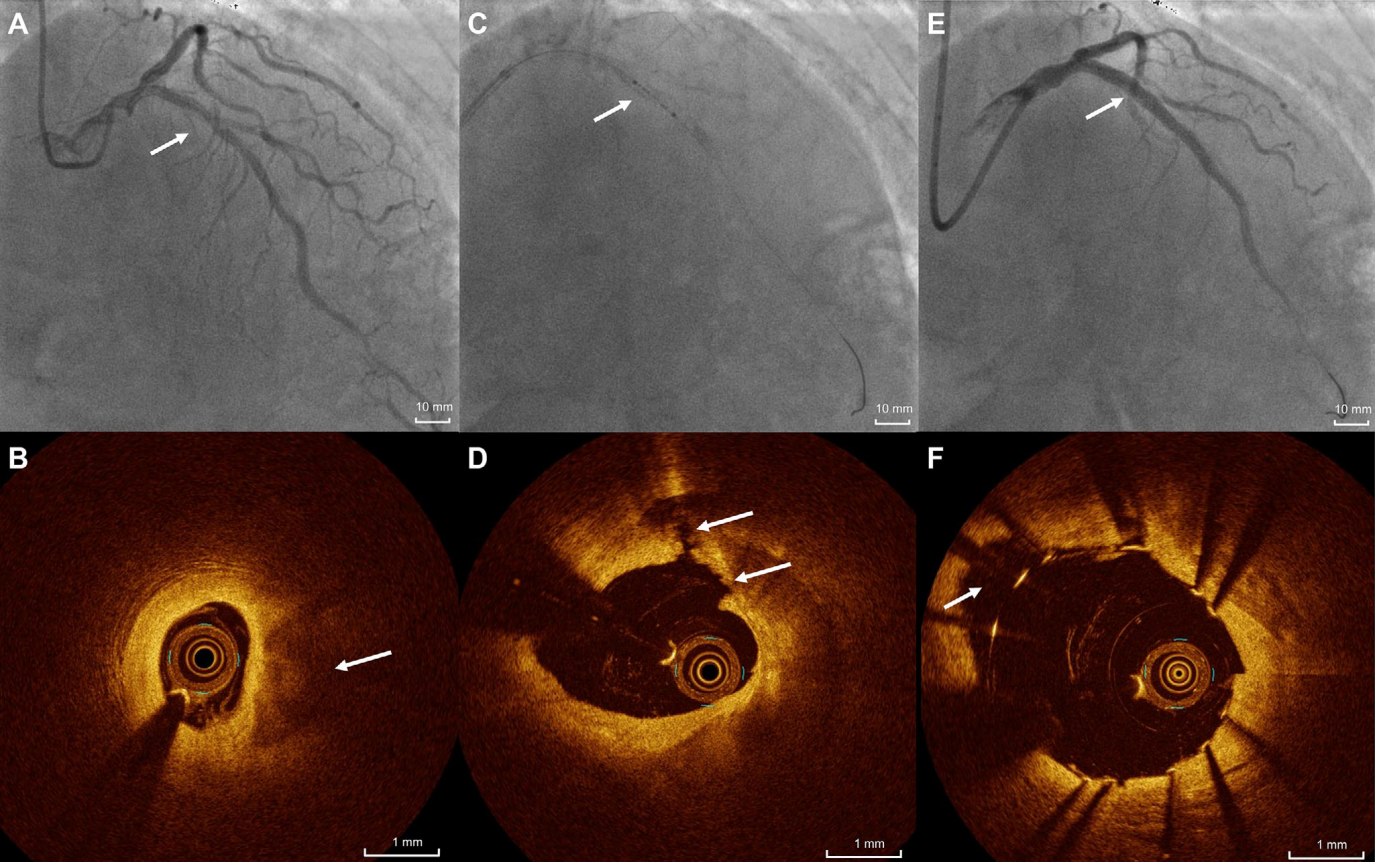
Images of a patient in the provisional IVL group. (A) Angiogram showing a stenosis with calcification in the left anterior descending artery (arrow). (B) OCT images acquired before IVL show heavy calcification in the stenotic calcified lesion (arrow). (C) IVL in the calcified lesion (arrow). (D) OCT images acquired after IVL show multiple calcium fractures and a large luminal gain (arrows). (E) Angiogram showing complete stent expansion (arrow). (F) OCT images obtained poststenting show full stent expansion and minimal malapposition, with an additional increase in the area of the acute gain (arrow). IVL, intravascular lithotripsy; OCT, optical coherence tomography. Scale bars correspond to 10 mm for angiogram images and 1 mm for OCT images.

**FIGURE 4:**
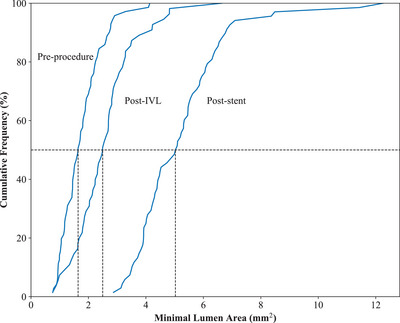
Cumulative frequency distribution curves demonstrating increased lumen area gain postintravascular lithotripsy (IVL) and poststent implantation by optical coherence tomography (OCT).

Among the 76 participants with available OCT data, typical cases of calcium fracture induced by IVL and stent expansion are illustrated in Figures 3 and S1. As detailed in Table 4 and Figure 4, preoperative measurements revealed a minimal lumen area (MLA) of 1.77 ± 0.72 mm^2^ and lumen stenosis of 76.15 ± 9.79%. At the site of maximum calcification, the calcification angle averaged 273.53 ± 77.63° and calcification thickness was 1.09 ± 0.34 mm. Following IVL, calcium fracture was observed in 76.8% of evaluable participants, with 41.1% exhibiting multiple fractures. Significant improvements were noted in lumen area and stenosis at all three measurement sites—MLA, maximum calcium site, and minimum stent area (*p *< 0.001 for all). The MLA increased to 2.59 ± 1.11 mm^2^, and stenosis was reduced to 66.06 ± 11.79%. Both the calcium angle and maximum calcium thickness also demonstrated significant reductions. Poststenting, these indicators further improved: the lumen area increased to 5.22 ± 1.69 mm^2^ (*p *< 0.001) and stenosis decreased to 32.39 ± 11.84% (*p *< 0.001). The immediate poststent lumen area gain was 3.48 ± 1.50 mm^2^, corresponding to a lumen gain rate of 219.58 ± 107.01%.

**TABLE 4:** OCT measurements and calcium fracture characteristics (*n* = 76).

	Time point			*p* Value		
OCT measurements (*n* = 76)	Pre‐IVL	Post‐IVL	Poststent	Pre‐IVL vs. Post‐IVL	Pre‐IVL vs. Poststent	Post‐IVL vs. Poststent
At MLA site						
Lumen area (mm^2^)	1.77 ± 0.72	2.59 ± 1.11	5.22 ± 1.69	<0.001	<0.001	<0.001
Area stenosis (%)	76.15 ± 9.79	66.06 ± 11.79	32.39 ± 11.84	<0.001	<0.001	<0.001
Calcium angle (°)	109.52 ± 89.96	93.23 ± 75.72	—	0.005	—	—
Max calcium thickness (mm)	0.71 ± 0.44	0.58 ± 0.40	—	0.003	—	—
Stent area (mm^2^)	—	—	5.15 ± 1.67	—	—	—
At max calcium site						
Lumen area (mm^2^)	3.50 ± 1.75	4.51 ± 1.59	7.08 ± 2.30	<0.001	<0.001	<0.001
Area stenosis (%)	53.32 ± 21.69	40.59 ± 15.94	8.18 ± 20.26	<0.001	<0.001	<0.001
Calcium angle (°)	273.53 ± 77.63	256.66 ± 74.66	—	<0.001	—	—
Max calcium thickness (mm)	1.09 ± 0.34	1.00 ± 0.26	—	0.013	—	—
Stent area (mm^2^)	—	—	6.59 ± 2.07	—	—	—
At final MSA site						
Lumen area (mm^2^)	2.97 ± 1.89	3.83 ± 1.81	5.39 ± 1.73	<0.001	<0.001	<0.001
Area stenosis (%)	62.99 ± 17.00	51.30 ± 15.05	30.23 ± 12.81	<0.001	<0.001	<0.001
Calcium angle (°)	157.34 ± 107.85	152.71 ± 102.86	106.04 ± 89.59	<0.001	<0.001	<0.001
Max calcium thickness (mm)	0.81 ± 0.47	0.76 ± 0.43	0.67 ± 0.43	0.046	0.008	0.016
Stent area (mm^2^)	—	—	5.03 ± 1.65	—	—	—
Calcium fracture characteristics						
Calcium fracture [*n* = 56]	—	43 (76.8)	—	—	—	—
No. of fractures	—		—	—	—	—
0 (%)		13 (23.2)				
1 (%)		20 (35.7)				
2 (%)		14 (25.0)				
≥3 (%)		9 (16.1)				
Maximum fracture depth (mm)	—	0.65 ± 0.29	—	—	—	—
Maximum fracture width (mm)	—	0.55 ± 0.37	—	—	—	—
Presence of coronary artery intramural hematoma (*n*, %) [*n* = 57]	—	3 (5.3)	—	—	—	—
Maximum angle of coronary artery intramural hematoma (°) [*n* = 3]	—	165.28 ± 79.15	—	—	—	—
Length of coronary artery intramural hematoma (mm) [*n* = 3]	—	10.60 ± 6.05	—	—	—	—
Other						
Poststent immediate lumen area gain (mm^2^)	—	—	3.48 ± 1.50	—	—	—
Poststent immediate lumen diameter gain (mm)	—	—	1.05 ± 0.38	—	—	—
Poststent immediate lumen gain rate (%)	—	—	219.58 ± 107.01	—	—	—

The numbers following the symbol “±” indicate SD (standard deviation).

*p* Values for longitudinal comparisons across time points were derived using paired *t*‐tests for normally distributed data or Wilcoxon signed‐rank tests for non‐normally distributed data, as determined by Shapiro–Wilk normality testing.

*Abbreviations*: OCT: optical coherence tomography; IVL: intravascular lithotripsy; MLA: minimal lumen area; MSA: minimum stent area.

## 3 Discussion

This prospective, multicenter, single‐arm **CO**ronary **CA**lcified Lesion **L**ithotripsy **P**rocedure (COCALP) trial represents the first investigation into the effectiveness and safety of this innovative IVL system for severely calcified coronary lesions in Chinese patients. The findings revealed a high procedural success rate of 97.4%, with the lower bound of the 95% CI exceeded the prespecified goal, thus meeting the effectiveness endpoint. Additionally, low rates of procedural complications and MACE were observed. OCT confirmed substantial calcium fracture following the IVL procedure, as well as significant improvements in lumen area and stenosis poststenting. These technological advancements reflect the broader “Panvascular Medicine+” paradigm, which emphasizes the integration of medicine and engineering [2]. “From doctors, by engineers/researchers, for patients” was advocated [2, 4]. The concept of “Panvascular Medicine+” represents an interdisciplinary approach that integrates medical knowledge with engineering innovation to address complex vascular diseases. It emphasizes the comprehensive management of all types of blood vessels and the use of advanced technologies to improve patient outcomes. Furthermore, the principle of “From doctors, by engineers/researchers, for patients” underscores the collaborative process of medical innovation. Clinicians identify clinical challenges, engineers and researchers develop solutions, and the ultimate goal is to enhance patient care. In this study, the development and application of the novel IVL system exemplify this collaborative approach, offering a promising solution for patients with severely calcified coronary lesions. Currently, severely calcified coronary lesions represent one of the last strongholds in the coronary field, making such innovation essential. This new IVL system is anticipated to be a promising treatment option for patients with severe coronary calcification.

Optimal preparation is essential prior to stent implantation in calcified coronary lesions. Recommendations for preparation include predilatation with noncompliant and high‐pressure balloons, the use of scoring or cutting balloons, or rotational atherectomy [20, 43, 44]. In line with previous studies, IVL offers a novel approach for preparing severely calcified coronary lesions [35, 40]. In this study, the Sonico‐CX balloon was employed for IVL. The effectiveness of this IVL system is substantiated by robust data analysis, including a comprehensive sample size calculation based on the PG derived from the DISRUPT CAD III trial [38]. Results confirmed that the study achieved its endpoint and validating the effectiveness of the IVL system. The procedural success rates, using a cutoff of residual stenosis at 30%, reported in previous studies such as the DISRUPT CAD series, particularly DISRUPT CAD III [38], and the CALCI‐CRACK trial [41], were 92.2 and 95.04%, respectively. A recent meta‐analysis, which used a broader definition with a cutoff of residual stenosis at <50% and included eight studies with 980 patients (1011 lesions), reported a success rate of 95.4% (95% CI: 92.9–97.9%) [45]. The IVL system utilized in this study achieved a procedural success rate of 97.4%, potentially surpassing historical benchmarks. This elevated success rate might be attributable to the system's effective calcium fracturing capabilities, which facilitate stent implantation and ultimately enhance lumen area and reduce stenosis.

The study population in this trial was largely comparable to that of previous studies, with a few minor differences. The percentage of male patients in the present study was 62%, which is somewhat lower than the 71.8–80.0% reported in earlier studies [19, 38, 40]. The primary target vessel for IVL was the LAD, aligning with findings from previous research [19, 35, 38]. The minimum vessel diameter (0.89 ± 0.36 mm) and diameter stenosis (68.7 ± 11.5%) in this study were generally consistent with those reported in the DISRUPT CAD studies [40]. In contrast to prior studies, where more than 36.4% of cases involved femoral access [38, 40], only 12 out of 266 cases in this study utilized femoral access. It has been suggested that combining trans‐radial access with IVL might be particularly synergistic [40]. It has been recognized that trans‐radial access is associated with a lower rate of MACE following PCI compared with femoral access [46, 47]. Thus, the higher proportion of trans‐radial access in this study and the lower complication rate might indicate more favorable outcomes with radial access for IVL. Additionally, the diameter of the lithotripsy balloon was closely aligned with the reference vessel diameter in this study, indicating that the 1:1 ratio for the novel IVL balloon was appropriate. The DISRUPT CAD III and CALCI‐CRACK trials also targeted a 1:1 balloon‐to‐vessel diameter ratio. However, the availability of seven sizes for the Sonico‐CX balloon catheter enhances its ability to match the reference vessel diameter, potentially contributing to a higher success rate.

Regarding vessel dissection, only two cases of F‐type dissection occurred post‐IVL. This rate is notably lower than the 13% reported in the initial IVL report [35], indicating an improvement in technique over time. Both cases of F‐type dissection were effectively managed. Other post‐IVL complications, including slow flow and no‐flow conditions, were also promptly resolved with appropriate intervention. A meta‐analysis of historical IVL reports has documented dissection rates of 0.5%, perforation rates of 0.4%, and MACE rates of 4.9% [45]. In contrast, the present study observed only six cases of 30‐day MACE (2.3%), which is relatively favorable. One cardiac death occurred within 30 days. It is speculated that the cardiac death was due to stent thrombosis. During the implementation of this project, the global COVID‐19 pandemic led to a significant increase in AEs among enrolled subjects. Despite of 15 device‐related AEs, the category with the highest proportion of AEs was infections, with COVID‐19 infections accounting for 13.91%, and other infections such as respiratory tract infections and urinary system infections also reported. Overall, this novel IVL system demonstrates a favorable safety profile with relatively low rates of MACE.

## 4 Limitations

This study has several limitations. First, participants were selected based on angiographic assessment of calcification rather than intraluminal imaging, which may have led to the exclusion of patients with severely calcified lesions that were not adequately visualized. Second, as a single‐arm study without a control group, it does not provide comparative data. This study is the first research in China on this type of device. Therefore, we choose the single‐arm trial. In the future, randomized trials with two groups in multicenter are needed. In real‐world settings, patients’ clinical conditions and procedural complexities may vary, highlighting the need for further evaluation of this IVL system's clinical performance in future studies conducted in more diverse and practical settings. Furthermore, the limited use of OCT (28.6%, 76 out of 266 patients) may introduce selection bias, as its application depended on operator judgment, lesion characteristics, patient consent, cost, and resource availability. While major endpoints were angiography‐ and clinically defined, the restricted OCT use may limit insights into IVL mechanisms and calcium morphology changes. Additionally, the assessment of calcification severity relied primarily on angiographic evaluation, which may not fully capture the complexity and heterogeneity of calcified lesions. Due to resource limitations, not all patients underwent intravascular imaging (intravascular ultrasound or OCT) or coronary computed tomography angiography (CCTA), and thus, a numerically quantified calcification score (e.g., Agatston score or calcium arc/length measurements) was not incorporated into our analysis. Future studies that utilize advanced imaging modalities to quantify calcification severity more precisely and broaden OCT implementation will provide a deeper understanding of calcification characteristics, validate our findings, and reduce bias.

## 5 Conclusion

This novel IVL system has a high success rate in severely calcified coronary lesions and with low procedural complication and MACE rates. This IVL system facilitates stent delivery and optimizes stent expansion. This new IVL system is expected to be a promising option for patients with severely calcified coronary lesions.

## 6 Materials and Methods

### 6.1 Study Design and Patients

The COCALP study was a prospective, multicenter, single‐arm trial designed to investigate the effectiveness and safety of this IVL system developed by Spectrumedics Medical Technology (Shanghai) Co., Ltd. (Shanghai, China) to treat severely calcified coronary lesions before stenting. It was conducted from December 2021 to May 2023 at 11 medical centers across China. This trial was registered in Chinese Clinical Trial Registry (ChiCTR; http://www.chictr.org.cn) under registration number ChiCTR2300073280. The principal investigators and study chair were granted full data access and took responsibility for verifying data accuracy and ensuring the report's alignment with the study protocol. The research protocol received ethical approval from the Institutional Review Board of Zhongshan Hospital, Fudan University (Approval No. 2021–120R), as well as the ethics review boards of all participating centers. The study adhered to the ethical principles outlined in the Declaration of Helsinki. All patients provided written informed consent before participating in the study.

This study included adult participants aged 18 years or above who were referred to the catheterization laboratory due to clinical indications such as stable angina, unstable angina, or silent ischemia, or based on noninvasive findings (e.g., positive stress test or CCTA suggesting calcified lesions). During the coronary angiography procedure, the operator assessed the target lesions for severe calcification, which was defined as the presence of radiopaque densities visible on fluoroscopy without cardiac motion prior to contrast injection, involving both sides of the arterial wall in at least two orthogonal angiographic views. The target lesions were ≤60 mm in length, with reference vessel diameters ranging from 2.5 to 4.0 mm. Patients were excluded if they had experienced an acute MI within 30 days prior to PCI or if their treatment involved the simultaneous use of rotational atherectomy or special balloons, such as chocolate, scoring, or spinous balloons. Those with CTO lesions, thrombus in the target vessel, target lesion located distal to the saphenous vein or left internal mammary artery/right internal mammary artery bypass grafts, or previous stent within 10 mm of the proximal or distal end of the target lesion were also excluded from the study. The final decision for enrollment was made during the coronary angiography procedure, ensuring that all included lesions strictly met the predefined criteria for severe calcification. The complete inclusion and exclusion criteria are detailed in the study registration available at ChiCTR. A subgroup of patients received OCT to evaluate the changes in calcium morphology after IVL procedure. The inclusion criteria for the OCT assessment were judged by the operator according to the coronary artery lesion situation, patient consent, procedural time, cost consideration, and operator preferences.

### 6.2 Angiography Assessment and Quantitative Coronary Angiography Quantification

During the coronary angiography procedure, the assessment of calcified lesions played a critical role in guiding the intervention. Preprocedurally, calcification distribution was evaluated using at least two orthogonal angiographic views, with the requirement that radiopaque densities were present on both sides of the arterial wall throughout the lesion. Lesion length and stenosis severity were also measured. Intra‐procedurally, real‐time angiography was utilized to guide the intervention, including wire crossing, balloon dilation, and device deployment, while simultaneously monitoring for complications such as dissection or impaired blood flow. Postprocedurally, OCT was employed to verify the efficacy of calcium modification; however, the initial screening and definition of severe calcification were exclusively based on angiographic findings. This comprehensive approach ensured accurate lesion characterization and procedural safety throughout the study.

Quantitative coronary angiography (QCA) was performed offline by an independent core laboratory using QAngio XA software (version 7.3.74.0; Medis Medical Imaging Systems, the Netherlands) for automated edge detection. Lesion morphology was evaluated based on established qualitative criteria. Key parameters analyzed included the minimum lumen diameter and residual stenosis percentage, which were utilized for primary efficacy endpoint assessment, along with the occurrence and classification of significant angiographic complications.

### 6.3 Procedures

The IVL procedure was performed using the Sonico‐CX balloon (Figure 1). To ensure that investigators adhere to the standard operating procedures (Table S4), all participating centers received training on the study protocol, device specifications, and usage methods at the project initiation. Additionally, during the first case enrollment, on‐site guidance was provided by the manufacturer's engineers. All operators were trained in the use of the device and were experienced interventional cardiologists with extensive PCI experience at their respective centers. The balloon catheter was deployed via a rapid exchange system using a 0.014‐in. guidewire. Of the seven available sizes, four (2.5 × 12, 3.0 × 12, 3.5 × 12, and 4.0 × 12 mm) were selected for use in this study. The IVL balloon, matched 1:1 to the reference vessel diameter, was advanced to the target lesion following guidewire passage. Initial balloon inflation was performed at 4 atm, delivering 10 impulses, followed by temporary inflation to 6–10 atm. If the lesion was not adequately predilated after the maximum number of pulses, an additional IVL catheter could be utilized. The IVL balloon position was adjusted, with overlapping applications as necessary, to ensure complete coverage of longer lesions. For patients in the OCT subgroup, OCT was conducted immediately before and after IVL, as well as poststent implantation, to provide a detailed characterization of the calcified lesions and to gain further insights into the underlying mechanisms of IVL.

### 6.4 Study Endpoints and Definitions

Procedural success served as the primary effectiveness endpoint, defined as stent implantation achievingwith ≤30% in‐stent residual stenosis without in‐hospital MACE, including cardiac death, MI, and target vessel revascularization. MI criteria adhered to the Fourth Universal Definition of MI [48].

Secondary effectiveness endpoints included angiographic success and device success. Angiographic success was defined as successful stent implantation with ≤30% in‐stent residual stenosis and absence of serious angiographic complications immediately after IVL, including type D to F severe dissection, perforation, acute occlusion, sustained slow‐flow, or no‐reflow. Device success was defined as the IVL catheter successfully crossing the target lesion and delivering lithotripsy without serious angiographic complications immediately following the procedure.

The safety endpoint included the incidence of MACE evaluated intraoperatively, prior to hospital discharge, at 30 days, and at 6 months, as well as the occurrence of other AEs.

### 6.5 Data Collection

Baseline and postprocedural coronary angiograms were digitally recorded and analyzed using QCA. All in‐hospital MACE were adjudicated by an independent clinical events committee, and recorded by Electronic Data Capture after discharge. Angiographic and OCT images were evaluated by an independent core laboratory (Clinical Consultancy Research Center Medtech (Shanghai) Co., Ltd). After discharge, follow‐ups were conducted at 30 days and 6 months post‐IVL.

### 6.6 Statistical Analysis

The sample size calculation was informed by the DISRUPT CAD III study [38], which involved a comparable patient population and similar primary endpoints, utilizing an objective PG. The PG for procedural success was established at 84%, with an anticipated procedural success rate of 90.2%. The procedural success rate was expressed as a point estimate with a 95% CI. The endpoint was considered achieved if the one‐sided lower limit of the 95% CI exceeded the PG. To meet this criterion, with an α value of 0.025 (one‐sided) and a *β* value of 0.2, a minimum sample size of 246 patients was required. Accounting for an 8% loss rate, the final sample size was determined to be 266 patients.

The FAS comprised participants determined according to the intention‐to‐treat principle, encompassing all participants who were enrolled in the study and received the treatment by investigational product. FAS was utilized for the assessment of baseline characteristics and effectiveness outcomes. For missing data related to the primary effectiveness endpoint in FAS, a worsts observation carried forward method was used, with missing data considered as procedural failure. The PPS was a subset of the FAS, comprising subjects with good adherence to the study protocol without major deviations. In PPS, no imputation was performed for missing data. The SS included all participants who were enrolled, received the treatment by investigational product, and had at least one safety evaluation, serving as the basis for safety assessments.

Categorical variables were presented as frequencies (*N*) and percentages (%). Continuous variables were evaluated for normality using the Shapiro–Wilk test and reported as mean ± standard deviation (mean ± SD). For normally distributed continuous variables in paired samples, paired t‐tests were employed; for non‐normally distributed paired continuous variables, Wilcoxon signed‐rank tests were used. A logistic regression model was constructed with procedural success as the outcome variable, reporting odds ratios (OR) with corresponding 95% CIs. The Clopper–Pearson method was utilized to calculate 95% CIs for overall rates. All tests were two‐sided, with a significance level set at *α* = 0.05; *p* values <0.05 were considered statistically significant. All analyses were performed using R software (Version 4.3.2) and SPSS version 25.0 (IBM Corp., Armonk, NY, USA).

## Author Contributions

Junbo Ge, Juying Qian, and Hao Lu conceived and supervised the study. Guosheng Fu, Genshan Ma, Xuebo Liu, Bei Shi, Jianfang Luo, Jingfeng Wang, Zhixiong Zhong, Hanbin Cui, Likun Ma, Juying Qian, Jian'an Wang, Hao Lu, and Junbo Ge designed the experiments. Xin Deng, Yiqing Hu, Guosheng Fu, Genshan Ma, Xuebo Liu, Bei Shi, Jianfang Luo, Jingfeng Wang, Zhixiong Zhong, Hanbin Cui, Likun Ma, Juying Qian, Jian'an Wang, Hao Lu, and Junbo Ge performed the experiments. Xin Deng, Yiqing Hu, and Hao Lu analyzed data. Xin Deng, Yiqing Hu, and Hao Lu wrote the manuscript. Junbo Ge, Hao Lu, Xin Deng, and Yiqing Hu made manuscript revisions. All authors reviewed the results and approved the final version of the manuscript.

## Conflicts of Interest

The Spectrumedics Medical Technology (Shanghai) Co., Ltd. (Shanghai, China) provided the hydroelectric shock wave lithotripsy instruments as well as necessary funding and technical support for this study, all of which were conducted in strict adherence to the clinical trial protocol and ethical guidelines. However, Spectrumedics Medical Technology (Shanghai) Co., Ltd. (Shanghai, China) had no role in the study design, data collection, analysis, interpretation, or writing of the manuscript.

## Ethics Statement

The **CO**ronary **CA**lcified Lesion **L**ithotripsy **P**rocedure (COCALP) study (No. ChiCTR2300073280) was a prospective, multicenter, single‐arm trial. The study was approved by the institutional review board of Zhongshan Hospital's medical ethics committee (Approval No. 2021–120R) and each participating center, and conducted in accordance with the Declaration of Helsinki. All patients have signed informed consent. All methods were performed in accordance with the relevant guidelines and regulations.

## Supporting information



Supporting Information

## Data Availability

The data that support the findings of this study are available on request from the corresponding author. The data are not publicly available due to privacy or ethical restrictions.
