# Supplementary material for: A Novel Intravascular Lithotripsy System in Severely Calcified Coronary Lesions: The Prospective COronary CAlcified Lesion Lithotripsy Procedure (COCALP) Study
Source: MedComm (2020). 2025 May 20;6(6):e70208. doi: 10.1002/mco2.70208 (PMC12092975; doi:10.1002/mco2.70208)
Supplement: Supplementary file 1 — Supporting Information [file MCO2-6-e70208-s001.docx]

Supplementary Materials for

**A novel intravascular lithotripsy (IVL) system in severely calcified coronary lesions: the prospective COronary CAlcified Lesion Lithotripsy Procedure (COCALP) study**

Xin Deng^1,#^, Yiqing Hu ^1,#^, Guosheng Fu^3^, Genshan Ma^4^, Xuebo Liu^5^, Bei Shi^6^, Jianfang Luo^7^, Jingfeng Wang^8^, Zhixiong Zhong^9^, Hanbin Cui^10^, Likun Ma^11^, Juying Qian^1^, Jian’an Wang^2^, Hao Lu ^1,^*, Junbo Ge^1,^*

^1^ State Key Laboratory of Cardiovascular Diseases, Department of Cardiology, Zhongshan Hospital, Fudan University, Shanghai Institute of Cardiovascular Diseases; National Clinical Research Center for Interventional Medicine, Shanghai, China;

^2^ Department of Cardiology, The Second Affiliated Hospital of Zhejiang University School of Medicine, State Key Laboratory of Transvascular Implantation Devices, Heart Regeneration and Repair Key Laboratory of Zhejiang Province, Transvascular Implantation Devices Research Institute, Hangzhou, China;

^3^ Department of Cardiology, Key Laboratory of Cardiovascular Intervention and Regenerative Medicine of Zhejiang Province, Sir Run Run Shaw Hospital, Zhejiang University School of Medicine, Hangzhou, China;

^4^ Department of Cardiology, Zhongda Hospital, Southeast University School of Medicine, Nanjing, China;

^5^ Department of Cardiology, Shanghai Tongji Hospital, Tongji University School of Medicine, Shanghai, China;

^6^ Department of Cardiology, Affiliated Hospital of Zunyi Medical University, Zunyi, China;

^7^ Department of Cardiology, Guangdong Cardiovascular Institute, Guangdong Provincial People's Hospital, Guangdong Academy of Medical Sciences, Guangzhou, China;

^8^ Department of Cardiology, Sun Yat-sen Memorial Hospital, Sun Yat-sen University, Guangzhou Key Laboratory of Molecular Mechanism and Translation in Major Cardiovascular Disease; Laboratory of Cardiac Electrophysiology and Arrhythmia in Guangdong Province, Guangzhou, China;

^9^ Center for Cardiovascular Diseases, Meizhou People’s Hospital (Huangtang Hospital), Meizhou Hospital Affiliated to Sun Yat-sen University; Guangdong Provincial Engineering and Technology Research Center for Molecular Diagnostics of Cardiovascular Diseases; Guangdong Provincial Key Laboratory of Precision Medicine and Clinical Translational Research of Hakka Population, Meizhou, China;

^10^ Key Laboratory of Precision Medicine for Atherosclerotic Diseases of Zhejiang Province, The First Affiliated Hospital of Ningbo University; Cardiology Center, The First Affiliated Hospital of Ningbo University; Ningbo Clinical Research Center for Cardiovascular Disease, Ningbo, China;

^11^ Department of Cardiology, The First Affiliated Hospital of USTC, Division of Life Sciences and Medicine, University of Science and Technology of China, Hefei, China.

# These authors contributed equally.

*** Correspondence should be addressed to:**

Prof. Hao Lu, M.D., Ph.D. (lu.hao@zs-hospital.sh.cn);

Prof. Junbo Ge, M.D., Ph.D. (jbge@zs-hospital.sh.cn).

Address: #1609 Xietu Road, Xuhui District, Shanghai 200032, CHINA.

**Table S1. Specifications of Sonico-CX & SHOCKWAVE C2 balloon catheter.**

|  | **Specifications** | **Nominal balloon diameter, mm** | **Nominal balloon length, mm** | **Working length, mm** | **Catheter compatibility** | **Max number of pulses** | **Crossing profile, inch** |
| --- | --- | --- | --- | --- | --- | --- | --- |
| **Sonico-CX** | 25012 | 2.5 | 12 | 1400 | 0.014-inch guidewire | 120 | 0.042 |
|  | 27512 | 2.75 |  |  |  |  |  |
|  | 30012 | 3.0 |  |  |  |  |  |
|  | 32512 | 3.25 |  |  |  |  | 0.044 |
|  | 35012 | 3.5 |  |  |  |  |  |
|  | 37512 | 3.75 |  |  |  |  |  |
|  | 40012 | 4.0 |  |  |  |  | 0.046 |
| **SHOCKWAVE C2** | 25012 | 2.5 | 12 | 1380 | 0.014-inch guidewire | 80 | 0.044 |
|  | 30012 | 3.0 |  |  |  |  | 0.045 |
|  | 35012 | 3.5 |  |  |  |  |  |
|  | 40012 | 4.0 |  |  |  |  | 0.047 |

**Table S2. Angiographic characteristics evaluated by QCA**

| **Characteristics** | **Value (n=264)^*^** |
| --- | --- |
| Target vessel |  |
| LM, n (%) | 10 (3.8) |
| LAD, n (%) | 193 (73.1) |
| LCX, n (%) | 8 (3.0) |
| RCA, n (%) | 52 (19.7) |
| Others, n (%) | 1 (0.4) |
| Eccentric, n (%) | 253 (95.8) |
| Bend, n (%) | 44 (16.7) |
| Tortuosity, n (%) | 27 (10.2) |
| Bifurcation, n (%) | 72 (27.3) |
| Reference vessel diameter, mm | 2.82±0.38 |
| Minimum vessel diameter, mm | 0.89±0.36 |
| Stenosis, % | 68.7±11.5 |
| Lesion length, mm | 35.0±15.6 |
| Calcified length, mm | 33.9±13.9 |

*Two patients did not have data available for QCA (one withdrew intraoperatively, and the other did not receive stent implantation), were not included in the QCA analysis.

The numbers following the symbol "±" indicate SD (standard deviation).

*LM: left main artery; LAD: left anterior descending artery; LCX: left circumflex artery; QCA: quantitative coronary angiography; RCA: right coronary artery; IVL: intravascular lithotripsy.*

**Table S3. Procedural characteristics (FAS)**

| **Characteristics** | **Value** |
| --- | --- |
| **Characteristics recorded in EDC** | **n=266** |
| Radial access, n (%) | 252 (94.7) |
| 6F guiding catheter, n (%) | 243 (91.4) |
| Post-IVL balloon dilatation, n (%) (n=265) | 90 (34) |
| Diameter of post dilatation balloon, mm | 2.53±0.49 |
| Pressure of post dilatation balloon, atms | 20±3.2 |
| Post-stent dilatation, n (%) (n=265) | 258 (97.4) |
| Postoperative hospital stays, days (n=265) | 2.2±1.8 |
| Diameter of lithotripsy balloon, mm | 2.81±0.33 |
| Number of total pulses, n | 39.9±23.3 |
| **Characteristics evaluated by QCA** | **n=264** |
| Post-IVL minimum vessel diameter, mm (n=198) | 1.48±0.38 |
| Total stent length, mm | 40.6±15.6 |
| Stents diameter, mm | 2.91±0.32 |
| Post-stent residual stenosis, % | 11.5±7.4 |
| With available OCT data, n (%) | 76 (28.6) |

The number of patients in each statistical analysis was 266 if not indicated specifically. Two patients did not have data available for QCA (one withdrew intraoperatively, and the other did not receive stent implantation), were not included in the QCA analysis.

The numbers following the symbol "±" indicate SD (standard deviation).

*EDC:* *electronic data capture; FAS:* *full analysis set; IVL: intravascular lithotripsy; OCT: optical coherence tomography.*

**Table S4. Sonico-CX Device Operation SOP.**

| No. | Step | Operation |
| --- | --- | --- |
| 1 | Device Preparation | - Connect the device to the power source and turn on the power switch. - Connect the foot pedal and catheter connection cable to the main unit. |
| 2 | Catheter Selection | - Reference vessel diameter: Catheter diameter = 1:1. If 1:1 size balloon is not available, it is recommended to use the nearest larger diameter balloon. |
| 3 | Balloon Preparation | - Draw 5 ml 1:1 mixture of saline and contrast solution into a syringe or inflation device. It is recommended to use a new syringe or inflation device to prevent blood contamination. - Connect the syringe or inflation device to the inflation port of the catheter. Apply negative pressure to remove air, repeating this process at least three times to ensure all air is purged. - Remove the protective needle and take off the balloon protective sheath. - Soak the balloon and the distal part of the catheter in saline for 15 seconds to activate the hydrophilic coating. - Cover the catheter connection cable with a protective sleeve. - Connect the catheter to the catheter connection cable. - Activate the treatment mode. |
| 4 | Lesion Preparation | - A pre-dilation balloon can be used to pre-dilate the target lesion to facilitate the delivery of the shockwave balloon. |
| 5 | Lesion Treatment | - Advance the catheter along the 0.014-inch guidewire to the target lesion. - Inflate the catheter to 4 atm and press the foot pedal to release pulses. After 10 consecutive pulses, the system will automatically pause for 10 seconds. At this point, inflate the balloon to 6-10 atm and observe the treatment effect. Alternatively, the operator can press the foot pedal intermittently to release pulses as needed based on the lesion condition. - Deflate the balloon to restore blood flow for 30 seconds. - Repeat the above steps for the second cycle of pulse release until the lesion is adequately treated. - If the lesion is long, move the catheter to repeat the treatment using the same method. It is recommended to overlap the treatment area by 2 mm to avoid untreated segments. - The recommended maximum number of pulses for overlapping treatment segments is 120. |

**Figure S1. Images of a patient in the provisional IVL group.**


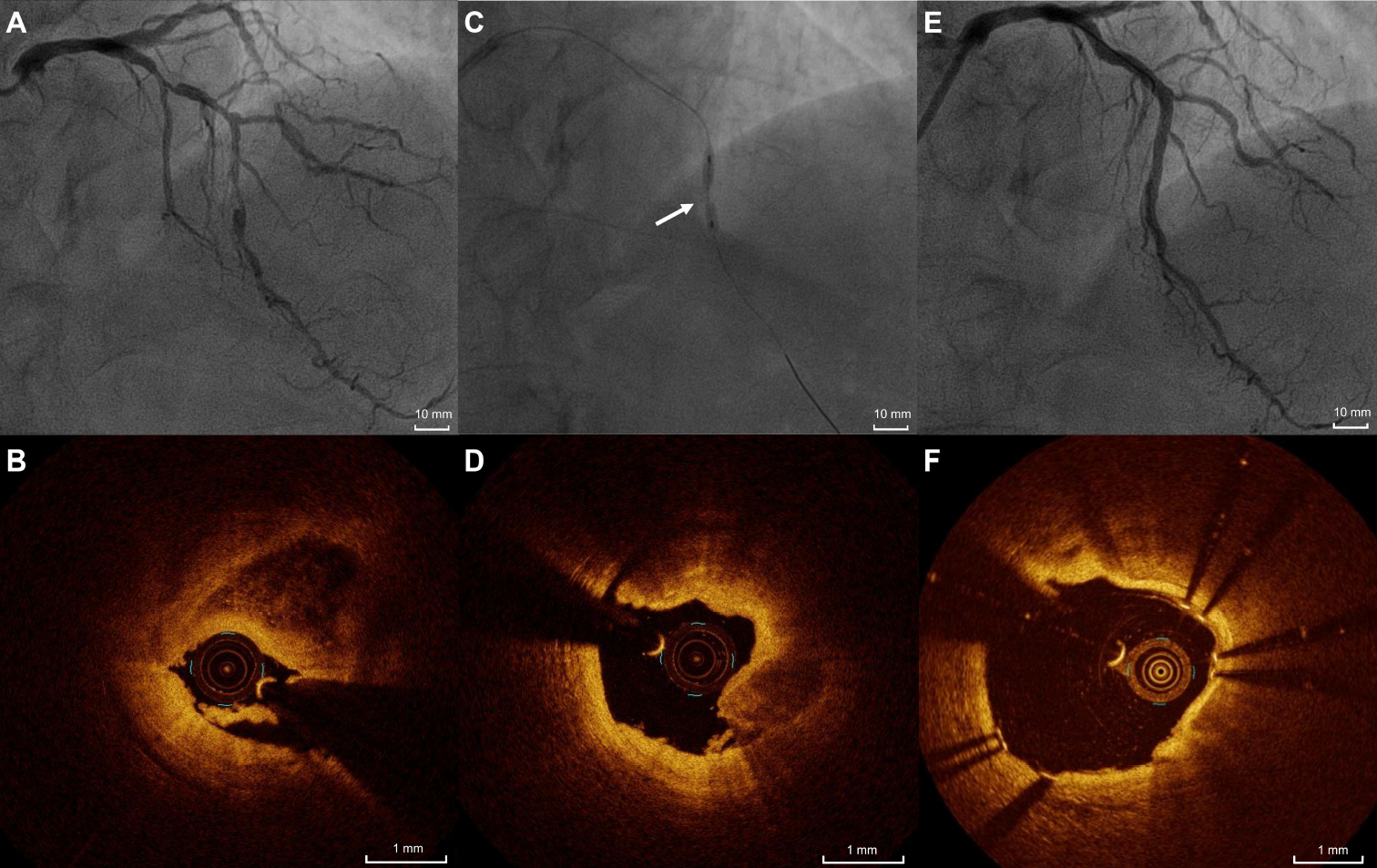


(A) Angiogram showing a stenotic calcified lesion in the left anterior descending artery. (B) OCT images acquired before IVL showing severe calcification in the stenotic calcified lesion. (C) The severe calcification led to underexpansion of the balloon (arrow). (D) OCT images obtained after IVL showing an acute luminal gain. (E) Angiogram showing complete stent expansion. (F) OCT images obtained post-stenting show full stent expansion and minimal malapposition. *IVL, intravascular lithotripsy; OCT, optical coherence tomography. Scale bars correspond to 10mm for angiogram images and 1mm for OCT images.*
